# Molecular Bases for the Regulation of NKG2D Ligands in Cancer

**DOI:** 10.3389/fimmu.2014.00106

**Published:** 2014-03-21

**Authors:** Leticia Huergo-Zapico, Andrea Acebes-Huerta, Alejandro López-Soto, Mónica Villa-Álvarez, Ana Pilar Gonzalez-Rodriguez, Segundo Gonzalez

**Affiliations:** ^1^Department of Functional Biology, University Institute of Oncology (IUOPA), University of Oviedo, Oviedo, Spain; ^2^Department of Hematology, Hospital Universitario Central de Asturias, Oviedo, Spain

**Keywords:** NKG2D, MICA, MICB, ULBP, NK cell, T cells, signaling pathways, regulation

## Abstract

NKG2D is an activating receptor expressed by NK and T cells primarily involved in the elimination of transformed and infected cells. NKG2D ligands are self-proteins restrictedly expressed in healthy tissues, but induced in response to signaling pathways commonly associated with transformation. Proliferative, tumor suppressor, and stress signaling pathways linked to the tumorigenic process induce the expression of NKG2D ligands, initiating an immune response against the incipient tumor. Nevertheless, the activity of NKG2D ligands is counter-regulated *in vivo* by the immunoediting of cancer cells, resulting in the expression of multiple mechanisms of immune evasion in advanced tumors. The redundancy of NKG2D ligands, besides increasing the complexity of their regulation, may impair the generation of these immune evasion mechanisms. In this review, we attempt to integrate the mechanisms and pathways involved in the regulation of NKG2D ligand expression in cancer.

## Human NKG2D and its Ligands

NKG2D is a type-II transmembrane-anchored glycoprotein constitutively expressed on the surface of NK, CD8 T, and γδ T cells (1). At the cell membrane, human NKG2D associates with DAP10, while mouse NKG2D may associate with both DAP10 and DAP12, which activate downstream signaling pathways resulting in the activation of NK cells and co-stimulation of CD8 T cells (2). Cross-linking of NKG2D receptor alone on freshly isolated NK cells does not seem to trigger a significant cell-mediated cytotoxic response; and simultaneous cross-linking of other activating receptors or stimulation of NK cells with IL-15 or high dose of IL-2 is rather necessary to kill tumor target cells (3).

**Figure 1:**
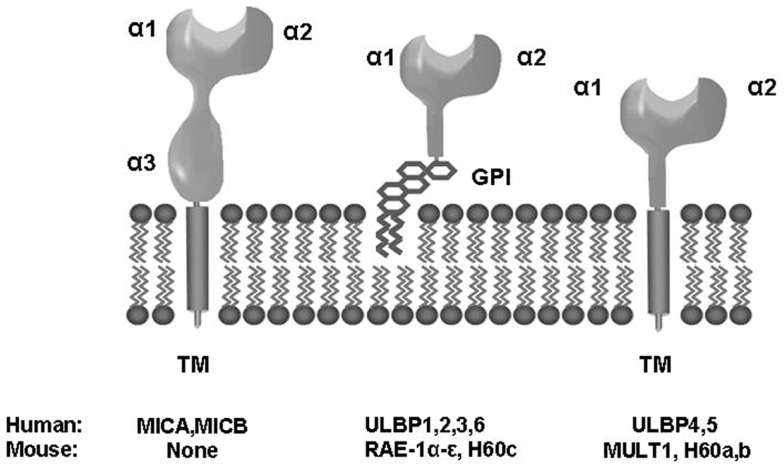
**Biochemical and structural properties of human and mouse NKG2D ligands**. TM, transmembrane; GPI, glycosylphosphatidylinositol.

NKG2D is a receptor for multiple ligands (NKG2DLs), which are distant members of the MHC class I family. In humans, NKG2D is a receptor for MICA and MICB (MICA/B) and ULBP1–6 molecules (4–8) (Figure 1). NKG2DLs are restrictedly expressed in healthy cells avoiding autoimmunity, but they are frequently over-expressed in infected and transformed cells, acting as a danger signal that favors the perforin-mediated elimination of transformed cells, thus acting as an extrinsic mechanism of cancer surveillance that complements the intrinsic processes that control growth and proliferation of tumor cells.

## NKG2D is Involved in the Immune Surveillance of Cancers

NKG2D-deficient mice are more prone to develop cancer (9), and neutralization of NKG2D increases the sensitivity of mice to carcinogen-induced sarcomas (10). Conversely, murine NK cells may efficiently eliminate cancer cells expressing NKG2DLs *in vivo*, highlighting the relevant role of this receptor in the cancer immune surveillance in mice (11–13). In humans, NKG2DLs are restrictedly expressed in healthy cells, but a broad expression of these proteins is observed in transformed cells, rendering them more susceptible to NK cell-mediated killing (1, 14–16). Moreover, cell-associated NKG2DL expression correlates with the outcome in colorectal (16, 17), breast, (18) and pancreatic cancers (19) and hepatocellular carcinoma (20). However, it is noteworthy that high expression of ULBP2 and ULBP4 has been paradoxically associated with poor prognosis in ovarian cancer, and it does not appear to be related to an increase of the shedding of these molecules (21, 22). Further, the development of immune evasion mechanisms by cancer cells has been associated with poor prognosis in different types of cancer (see below). Thus, the serum level of soluble MICA (sMICA) is an independent prognostic factor for multiple myeloma (23) and advanced hepatocellular carcinoma (24). sMICA and soluble MICB (sMICB) serum levels were correlated with disease stage and survival rates in oral squamous cell carcinoma patients (25), whereas soluble ULBP2 (sULBP2) was associated with poor prognosis in melanoma (26), chronic lymphocytic leukemia (27), and lung cancer (28).

Albeit these data clearly indicate an important role for the NKG2D response in the immune surveillance of human cancers, the molecular bases underlying the regulation of NKG2DL expression have not been fully elucidated. Nevertheless, increasing evidence indicates that the activation of oncogenic pathways commonly associated with the tumorigenic process may be responsible for the induction of NKG2DL expression and the activation of the NKG2D response (29) (Table 1).

**Table 1:** **Main signaling pathways that regulate NKG2D ligand expression**.

Signaling pathway	Cell model	NKG2D ligand	Reference
**STRESS PATHWAYS**			
DNA damage response	Non-tumor cell lines	Mouse and human ligands	(30)
	Myeloma		(31)
Thermal stress	Epithelial cell lines	MICA/B	(32, 33)
Oxidative stress	Epithelial cell lines/epithelial bronchial cells	MICA/B, ULBP1–4	(34, 35)
**PROLIFERATIVE/ONCOGENE PATHWAYS**			
c-Myc	Primary lymphoma	Rae-1ε	(36)
H-RasV12	Mice and human cell lines	Rae-1α Rae-1β, ULBP1–3	(37)
HER2/HER3	Breast cancer cell lines	MICA/B	(38)
BCR–ABL	CML cells	MICA/B	(39–41)
PI3K	Multiple	Mouse and human ligands	(37, 38, 42, 43)
**TUMOR SUPPRESSOR PATHWAYS**			
P53	Epithelial cell lines	ULBP1,2	(44–46)
**TUMOR PROGRESSION AND METASTASIS**			
EMT	Epithelial cell lines	MICA/B, ULBP1–3	(47)

*CML, chronic myelogenous leukemia; EMT, epithelial-to-mesenchymal transition*.

## DNA Damage and NKG2D Response

DNA damage potentially represents an initiating event for carcinogenesis as it is frequently observed in precancerous lesions. As a result of the DNA damage response (DDR), cell cycle checkpoints are activated, stopping the cell cycle to give the cell time to repair the damage, and, if such damage is too great, to trigger apoptosis. DNA damage checkpoint activation is controlled by two kinases: ATM (ataxia telangiectasia, mutated), which responds to DNA double-strand breaks and disruptions in chromatin structure; and ATR (ATM- and Rad3-related), which primarily responds to stalled replication forks. Activated ATM or ATR initiates a protein kinase cascade that includes the activation of the checkpoint kinases CHK1 and CHK2, and p53. In a seminal paper, it was shown that DNA damage also activates the immune response as an additional extrinsic control mechanism that favors the elimination of damaged and cancer cells through the induction of NKG2DL expression (30). It is worth-mentioning that the up-regulation of NKG2DLs occurs, at least in mice, in a p53-independent manner, which is particularly relevant given that a p53 expression is lost in a majority of clinical aggressive and invasive cancers. However, the downstream regulators of the DDR involved in NKG2DL expression remain ill-defined.

DNA damage response is activated in response to ionizing radiation and chemotherapy. Consequently, the immune response may contribute to the efficacy of these conventional cancer treatments. Many viruses with distinct replication strategies, such as DNA viruses and retroviruses, may also activate the DDR, which may potentially activate the NKG2D response. The replication of viral DNA in the nucleus has the potential to trigger the DDR if the ends of viral genomes are exposed and recognized as double stranded breaks. Retroviral DNA integration creates a discontinuity in the host cell chromatin, also activating the DDR. Likewise, induction of NKG2DLs by two retroviral infections, caused by Abelson murine leukemia virus and human immunodeficiency virus (HIV), supports a role of DDR in the NKG2D-mediated anti-viral response (48, 49). A more universal role of this pathway in the elimination of virus-infected cells remains to be established.

NKG2DL expression may also be induced as a result of DNA replication stress and activation of ATM/ATR in human T cells that proliferate in response to mitogens, antigens, and superantigens (50). This has been proposed to limit undesired immune responses; however, the underlying mechanisms and the biological significance of NKG2DL expression in T cells and other healthy cells are poorly understood.

## Other Types of Cellular Stress Regulate NKG2D Response

Different forms of cellular stress frequently associated with the tumorigenic process are involved in the regulation of NKG2DL expression. Early reports showed that heat shock was able to induce *MICA* and *MICB* expression (32). This induction of *MICA/B* transcription was lately described as the result of the binding of heat shock factor 1 (HSF1) to the promoters of *MICA* and *MICB* genes (33). Several studies showed that oxidative stress also induced *MICA/B* gene and protein expression in several tumor cell lines (34, 51). In normal human bronchial epithelial cells, oxidative stress induced MICA and ULBPs expression through the activation of ERK (35). Hypoxia has also been shown to down-regulate the surface expression of MICA in osteosarcoma cell lines through the induction of hypoxia-inducible factor 1α (HIF-1α), but independently of nitric oxide (NO) production (52).

## Proliferative Signals Induce NKG2D Response

Early reports showed that *MICA/B* mRNA and protein expression are mostly limited to proliferating epithelial cells, but they are scarcely expressed in quiescent cells (32, 33, 50). Recently, proliferation has been associated with the induction of the expression of murine RAE-1 family of NKG2DLs in mouse fibroblasts, and of MICA/B and ULBP2 in HCT116 colorectal cancer cells (42). The induction of NKG2DL expression was independent of stress pathways. Instead, *Raet1* genes, but not MULT1 and H60b, were found to be direct targets of E2F family of transcription factors, which plays a major role during the G1/S transition and cell cycle reentry. This provides a mechanistic link between proliferation and NKG2D response. Moreover, murine RAE-1ε induction in primary fibroblast cultures was blocked by inhibiting proliferative pathways such as cyclin-dependent kinases, PI3K–mTOR, and MAPK pathways (42).

Unrestrained proliferation is a common characteristic of cancer cells. Albeit it has not always been mechanistically linked to cell proliferation, it has been reported that inappropriate over-expression of several oncogenes induced NKG2DL expression independently of DDR. However, the expression of certain oncogenes (*K-ras* and *c-myc* or *Akt* and c-*myc*) was not sufficient to induce NKG2DL expression in primary ovarian epithelial cells (30). This probably reflects that transformation is a multistep process, and changes in many pathways, such as tumor suppressor pathways, are required for both transforming a normal cell into a cancer cell and for activating the NKG2D response. In fact, it has been reported that several oncogenic pathways up-regulate NKG2DLs. For instance, NKG2DLs are induced on spontaneously arising tumors in an Eμ-myc murine model of lymphoma (36). The induction of NKG2DLs involves the activity of c-Myc, which is a master regulator of proliferation. Similarly, the oncogenic H-RasV12 protein up-regulates the expression of Rae-1α and Rae-1β in mouse and of ULBP1–3 in human cells (37). H-RasV12 mainly regulated the expression of Rae-1 by post-transcriptional mechanisms via Raf–MAPK/MEK and PI3K pathways. Ras signals also contribute to the induction of NKG2DL expression in primary fibroblast cultures in response to the serum, which may be blocked by epidermal growth factor receptor (EGFR) inhibitors (37). EGFR comprises a family of four closely related transmembrane tyrosine kinase receptors (also known as HER1–4). Amplification or over-expression of the HER2/HER3 dimer is involved in the progression of different cancers, particularly in certain types of aggressive breast cancer. It has been shown that over-expression of HER2/HER3 activates the expression of MICA/B, mainly through the PI3K/AKT pathway (38). Philadelphia chromosome is a specific chromosomal translocation associated with chronic myelogenous leukemia (CML). It results in BCR–ABL fusion protein, with a continuously activated tyrosine kinase activity, thus promoting the unrestrained cell division of leukemia cells. BCR–ABL kinase activity was also linked to NKG2DL expression and the activity of NK cells (39, 40). Thus, BCR/ABL induces the MICA surface expression on CML leukemia cells, whereas it was absent on healthy hematopoietic cells (41).

Induction of host cell proliferation is a common viral strategy that favors viral survival and replication. However, it may also induce NKG2DL expression, resulting in an NKG2D-dependent elimination of the infected cell. Thus, the activation of PI3K pathway by the murine cytomegalovirus results in the expression of RAE-1 family of NKG2DLs (43). Similarly, adenovirus serotype 5 (Ad5) E1A oncogene up-regulates RAE-1 expression, but not murine ULBP-like transcript 1 (53).

Overall, these data suggest that proliferative signals generated by oncogene activation and virus infection and, possibly by other physiological situations, may alert the immune surveillance response through the induction of the NKG2D response.

## Tumor Suppression and Senescence may Induce NKG2DL Expression

The deregulation of the activity of oncogene proteins is counter-regulated by tumor suppressor proteins. These proteins repress cellular proliferation and the loss of their function is an additional characteristic associated with tumorigenesis. For instance, in the Eμ-myc murine model of lymphoma, sustained over-expression of Myc signaling induces p19^Arf^ expression, resulting in apoptosis through the inhibition of Mdm2 and stabilization of the p53 tumor suppressor protein. Thus, a combination of deregulated activity of Myc and the loss of a tumor suppressor protein (either p19^Arf^ or p53) is involved in both tumor progression and in the induction of Rae-1 in lymphoma cells (36). Thus, despite the existence of few experimental data, the current knowledge about tumor biology suggests that the combination of oncogenes and tumor suppressors may be involved in the regulation of NKG2DL expression.

Accordingly, the tumor suppressor p53 has been associated with the regulation of NKG2D response. Over-expression of wild-type p53 in cancer cell lines strongly up-regulated the expression of *ULBP1* and *ULBP2*, but not of other NKG2DLs, upon binding of p53 to response elements located in the intronic regions of these genes (44, 45). Moreover, the small molecule inhibitor Nutlin-3a decreased ULBP2 levels in a p53-dependent manner, through the increase in cellular levels of the suppressive miR-34 (46).

Additionally to growth arrest and apoptosis, p53 may induce senescence in response to diverse forms of cellular stress, including telomere shortening, DNA damage, oncogene activation, oxidative stress, and chemotherapeutic drug administration. The senescence program activates p53 and p16^INK4a^–pRb tumor suppressor pathways, which establish an irreversible form of cell cycle arrest. The most fully described function of senescence *in vivo* is the ability to establish a potent barrier to tumorigenesis in response to oncogene activation or DNA damage. A typical characteristic of senescent cells, known as “senescence-associated secretory phenotype,” is the secretion of inflammatory cytokines and chemokines that may recruit and activate distinct adaptive and innate immune cells subsets, including NK and T cells.

It has been shown that chemotherapeutic drug treatment of myeloma cells induces DDRs and activates the expression of NKG2DLs, mainly in senescent cells, resulting in their elimination by NK cells through the recognition by NKG2D and DNAM-1 receptors (31). NKG2DLs are also up-regulated on activated senescent hepatic stellate cells (54). Further, a recent study in a mouse model of senescence generated by induction of p53 expression also showed that NK cells participate in the elimination of senescent cells in an NKG2D-dependent manner (55). Nevertheless, induction of p53 did not increase NKG2DL expression, but, instead, induced senescent cell to secrete chemokines that recruit NK cells.

## NKG2DL Expression during Tumor Progression

It has been well established that NKG2D may protect the host from tumor initiation (9–13); however, less information exists regarding the role of NKG2D in tumor progression and metastasis. Early reports showed the loss of MICA/B expression in metastatic melanoma suggesting the immune selection of MICA/B negative tumors (56). Similarly, we have recently reported that epithelial-to-mesenchymal transition (EMT) is an immunological checkpoint that controls tumor progression through NKG2D-mediated immune responses (47, 57). EMT is one of the first steps of the metastatic cascade, which results in the loss of epithelial characteristics and the gain of mesenchymal properties by malignant cells, such as migration and invasiveness. EMT induction by Snail1 over-expression, GSK-3 inhibition, or TGF-β stimulation up-regulated NKG2DL expression in epithelial cells, rendering EMT cells more susceptible to NK cell-mediated killing. Sp1 and Sp3 transcription factors are key regulators of the basal transcription of *MICA/B* and *ULBP1–3* (33, 58, 59); and induction of Sp1 activity in colorectal tumor cells was involved in the up-regulation of NKG2DL expression during the EMT process. *In vivo*, MICA/B and ULBP1 proteins are expressed in healthy colon mucosa cells, displaying a polarized apical distribution; and no interaction with NKG2D-bearing immune cells was observed (47, 57). The polarized expression of MICA and ULBP1 was also observed in malignant cells retaining epithelial characteristics, whereas a loss of the polarization of NKG2DL expression was observed in malignant cells that suffer EMT. Moreover, a dramatic increase in the number of NKG2D-bearing tumor-infiltrating T lymphocytes was observed in specimens lacking MICA expression, suggesting that the loss of the epithelial integrity and polarity, characteristics of the EMT process, may allow the diffusion of MICA/B proteins along the membrane of mesenchymal cells, resulting in their elimination by NKG2D-bearing immune cells. Overall, these data suggest that tumor progression and metastasis, a key characteristic of malignant cancers, may also be under the control of the NKG2D response.

## Tumor Evasion of NKG2D Response

NKG2D-deficient mice expressed higher amounts of NKG2DLs in cancer cells than similar tumors in wild-type mice, which suggests that NKG2DL expression in a host may be counter-regulated by the immunoediting of cancer cells (9). This is in agreement with the description of a plethora of immune evasion mechanisms that impair the NKG2D-mediated response. Repression of NKG2DL expression is frequently observed in advanced tumors and metastases compared with primary tumors, and this may be regulated by epigenetic mechanisms. In advanced tumors, histone deacetylases (HDACs) may interact with the promoter region of NKG2DL genes, regulating the chromatin structure and impairing the access of transcription factors to their promoters. Thus, HDAC1 may inhibit the transcription of *MICA/B* on leukemia cells (60), whereas HDAC3 may repress the transcription of *ULBP1–3* in epithelial tumors (59). Consequently, HDAC inhibitors stimulate the expression of NKG2DLs on tumor cells (60–63). Other mechanisms, such as the over-expression of suppressive microRNAs in tumors, may also account for the repression of NKG2DL expression (46, 64–66). Contrarily, sustained NKG2DL expression and prolonged interaction with NKG2D receptor also leads to a strong down-regulation of NK cell cytotoxic activity (67, 68).

NKG2DLs may be also shed as soluble proteins (69, 70), which may cause the endocytosis and degradation of NKG2D receptor in immune cells (2). Interaction of MICA on the surface of tumor cells with the chaperon molecule ERp5 plays a relevant role in sMICA shedding (71). ERp5 may form a transitory disulfide bond with MICA, which induces a conformational change, allowing the proteolytic cleavage of MICA by proteases. Likewise, membrane ERp5 was functionally associated with sMICA shedding in chronic lymphocytic leukemia patients (72); and sMICA serum levels have been associated with ERp5 expression in multiple myeloma (73) and Hodgkin lymphoma (74). sULBP2 may also be released from tumor cells by proteolytic cleavage (75). However, engagement of sULBP2 not necessarily down-regulates NKG2D receptor. sULBP4 may be shed from tumor cells by alternative splicing mechanisms (76). Additionally, cancer cells may release MICA, MICB, and ULBPs into exosomes (77, 78). This event appears to be ligand-dependent, since ULBP2 is mainly shed by metalloproteinases, whereas ULBP3 is abundantly released as part of exosomes. Moreover, in the case of MICA, it may be allele-dependent (79). Exosomes containing NKG2DLs selectively down-regulate NKG2D expression on CD8 T and NK cells.

## Concluding Remarks

As discussed above, relevant biological pathways associated with tumorigenesis, including proliferative, tumor suppressor, and stress pathways, are key signals involved in the induction of the NKG2D response in cancer. Many of these pathways are frequently activated in viral infections and in other pathological and physiological situations, providing a common link in the regulation of NKG2DLs between these pathologies and cancer. Nevertheless, little information regarding to the regulation of NKG2DLs in non-cancer-conditions is available, and future research in this field is needed.

However, the activity of NKG2DLs is counter-regulated *in vivo* by the immunoediting of cancer cells and for the development of immune evasion mechanisms in virus. Likewise, advanced tumors develop multiple mechanisms of immune evasion that frequently modify NKG2DL expression, thus making difficult to differentiate the intrinsic mechanisms of NKG2DL regulation from the consequences of the immune selection. As described above, distinct NKG2DLs are differentially regulated by specific oncogenic pathways. Thus, the redundancy of NKG2DLs presumably increases the range of different pathological situations in which NKG2D response may be activated and may also impair the generation of immune evasion mechanisms.

## Author Contributions

All authors participated in the preparation, discussion, and writing of this manuscript.

## Conflict of Interest Statement

The authors declare that the research was conducted in the absence of any commercial or financial relationships that could be construed as a potential conflict of interest.
